# Roles of Lipid Metabolism in Pulmonary Hypertension: Friend or Foe?

**DOI:** 10.3390/biom15121679

**Published:** 2025-12-01

**Authors:** Wei Huang, Runxiu Zheng, Lijun Gong, Yu Zhang, Junlan Tan, Xianya Cao, Lan Song, Aiguo Dai

**Affiliations:** 1 School of Integrated Chinese and Western Medicine, Hunan University of Chinese Medicine, Changsha 410208, China; 20213306@stu.hnucm.edu.cn (W.H.); 20232134@stu.hnucm.edu.cn (R.Z.); yuzhang@stu.hnucm.edu.cn (Y.Z.); 2 Hunan Provincial Key Laboratory of Vascular Biology and Translational Medicine, Changsha 410208, China; l00412@hnucm.edu.cn (L.G.); junlantan@stu.hnucm.edu.cn (J.T.); 20183116@stu.hnucm.edu.cn (X.C.); songlan311492@hnucm.edu.cn (L.S.); 3 School of Medicine, Hunan University of Chinese Medicine, Changsha 410208, China

**Keywords:** pulmonary hypertension, lipid metabolism, fatty acid oxidation, metabolic reprogramming, therapeutic strategies

## Abstract

Pulmonary hypertension (PH) is a progressive cardiopulmonary disorder characterized by vascular remodeling and right ventricular (RV) failure. Recently, attention to lipid metabolism in PH has revealed multiple mechanisms that drive disease progression, including alterations in energy supply, oxidative stress, inflammatory signaling, and epigenetic regulation. Notably, lipid metabolism in PH exhibits marked spatiotemporal heterogeneity. This creates a therapeutic paradox in which the same metabolic intervention may exert opposing effects depending on tissue type and disease stage. Despite these challenges, targeting lipid metabolism remains an attractive therapeutic strategy. Preclinical and early clinical studies suggest that both small-molecule metabolic modulators and natural compounds hold promise for reversing pulmonary vascular remodeling and improving RV function. This review summarizes current advances in lipid metabolic reprogramming in PH and highlights the challenges of developing tissue- and time-specific interventions.

## 1. Introduction

The Seventh World Symposium on Pulmonary Hypertension redefined pulmonary hypertension (PH) as a mean pulmonary arterial pressure (mPAP) greater than 20 mmHg, determined by right heart catheterization [1]. PH is a progressive, life-threatening disorder characterized by persistent pulmonary vasoconstriction and vascular remodeling, ultimately leading to right ventricular (RV) failure and death [2]. Despite significant therapeutic advances in recent years, a substantial proportion of patients exhibit inadequate responses or poor tolerance to current treatments, and lung transplantation often remains the final option [3]. Among the clinical subtypes of PH, pulmonary arterial hypertension (PAH) is particularly severe, with data from the REVEAL Registry (The Registry to Evaluate Early and Long-term PAH Disease Management) showing 5-year survival rates of 72.2%, 71.7%, 60.0%, and 43.8% for patients with functional classes I–IV, respectively [4]. Because early diagnosis of PH relies mainly on invasive hemodynamic evaluation, and pathological vascular remodeling is difficult to detect at an early clinical stage, most patients are diagnosed at advanced stages, resulting in poor prognosis.

PH patients typically exhibit significant metabolic abnormalities, such as enhanced glycolysis [5] and disruptions in lipid and glutamine metabolism [6]. Metabolic reprogramming often induces profound changes in energy metabolism, signal transduction, and cellular structure, driving the progression of pulmonary and cardiac pathologies [7]. Fatty acids, as key components of membrane phospholipids and energy reserves, display abnormal utilization and accumulation during PH pathogenesis [8]. This dysregulation promotes adverse pulmonary vascular remodeling, whereas it induces lipotoxic deposition and functional decompensation in the RV [9]. Similar to cancer cells, pulmonary vascular cells increase fatty acid uptake and de novo synthesis to meet demands for rapid proliferation and resistance to apoptosis, whereas the RV commonly shows reduced fatty acid oxidation (FAO) and lipid droplet accumulation, resulting in energy crisis and lipotoxic injury. This “tissue-specific” metabolic alteration not only reveals the complexity of lipid metabolic reprogramming during PH progression but also suggests that precise interventions targeting lipid metabolism may emerge as novel therapeutic strategies. This review primarily examines how fatty acid metabolism contributes to the development and progression of pulmonary hypertension and considers its translational potential for therapy.

## 2. The Role of Lipid Metabolic Reprogramming in PH

Lipid metabolism plays a vital role in maintaining cellular energy homeostasis and normal cellular function under physiological conditions. However, these metabolic pathways are profoundly reprogrammed during the pathogenesis of PH, driving adverse vascular remodeling and right ventricular dysfunction. This section first provides an overview of the fundamental physiological regulatory mechanisms of lipid metabolism. Subsequently, we examine the pathological upregulation of lipid acquisition, driven by increased uptake and de novo synthesis. Finally, we specifically focus on the dysregulation of FAO as the most critical pathway for lipid utilization. This highlights the distinct roles of lipid metabolism in pulmonary vascular remodeling versus right ventricular failure.

### 2.1. Physiological Regulatory Mechanisms of Lipid Metabolism

**Figure 1:**
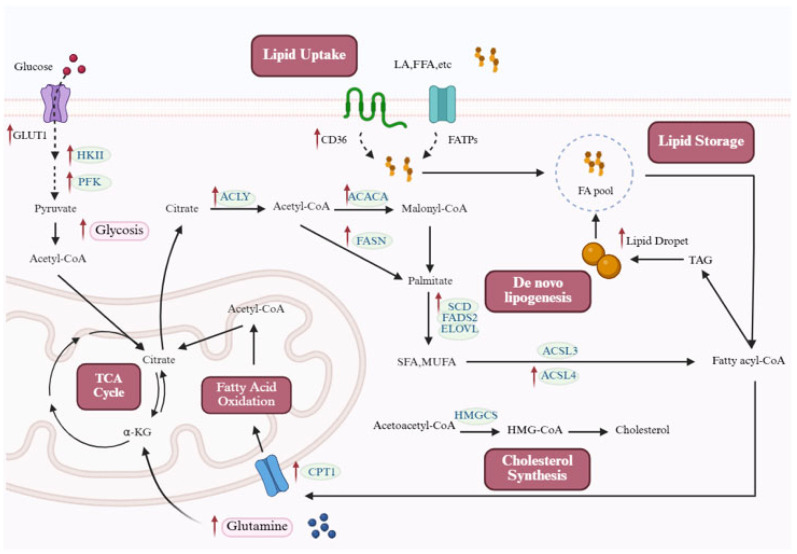
Lipid metabolic reprogramming in PH. Cellular lipid synthesis integrates multiple metabolic inputs. Fatty acids can be supplied through passive diffusion or protein-mediated transport (e.g., FATPs and CD36). In addition, the coordinated upregulation of glycolysis and glutaminolysis ensures a continuous supply of acetyl-CoA and citrate. Along with the acetyl-CoA generated from mitochondrial FAO, these sources converge in the TCA cycle to produce citrate. Exported citrate provides acetyl-CoA via ACLY, fueling both fatty acid and cholesterol biosynthesis. The rate-limiting step of lipogenesis is catalyzed by ACACA, producing malonyl-CoA, which, together with acetyl-CoA, is utilized by FASN to generate palmitate. This product can be further elongated by ELOVL or desaturated by SCD and FADS2 to form SFAs and MUFAs. Cholesterol synthesis begins with acetyl-CoA, which is processed by HMGCS through the mevalonate pathway. Lipids are stored in LDs in the form of TAGs, with DGAT catalyzing the final step of TAG synthesis. During the pathogenesis of PH, cells reprogram lipid metabolism to adapt to genetic alterations and environmental stress, as illustrated by the altered pathways and upregulated proteins highlighted in the figure. Red arrows in the figure denote the upregulated expression of target molecules in PH. ACACA: acetyl-CoA carboxylase; ACLY: ATP-citrate lyase; ACSL: acyl-CoA synthetase long-chain family; CD36: cluster of differentiation 36; CPT1: carnitine palmitoyltransferase 1; ELOVL: elongation of very long-chain fatty acid proteins; FADS2: fatty acid desaturase 2; FASN: fatty acid synthase; FATPs: fatty acid transport proteins; FFAs: free fatty acids; GLUT1: glucose transporter 1; HKII: hexokinase II; HMG-CoA: 3-hydroxy-3-methylglutaryl-CoA; HMGCS: 3-hydroxy-3-methylglutaryl-CoA synthase; MUFAs: monounsaturated fatty acids; PFK: phosphofructokinase; SCD: stearoyl-coA desaturase; SFAs: saturated fatty acids; TAGs: triacylglycerols; TCA: tricarboxylic acid. This figure was created with BioRender.com.

The physiological regulation of lipid metabolism is governed by the coordination of multiple core processes, including uptake, de novo synthesis, storage, and catabolism. Cellular uptake of exogenous fatty acids is primarily mediated by fatty acid transport proteins (FATPs) and cluster of differentiation 36 (CD36) [10,11,12,13]. In de novo lipid synthesis, glucose and glutamine serve as critical substrates (Figure 1). Glycolytic processing of glucose produces pyruvate, which then enters the mitochondria and is metabolized into citrate. The generated citrate is released into the cytosol, where it functions as a critical precursor for fatty acid and cholesterol synthesis. Glutamine (Gln) is the most abundant and versatile amino acid in the body. Intracellular Gln is deaminated by glutaminase to glutamate, which is subsequently converted to α-ketoglutarate (α-KG) by glutamate dehydrogenase. The process is essential for sustaining rapid proliferation in cancer cells and other dividing cell types [14,15,16,17]. Under hypoxic or mitochondrion-deficient conditions, glutamine-derived α-KG is converted to citrate through reductive carboxylation, which enters the cytosol and is transformed into acetyl-CoA, thereby promoting de novo lipid synthesis [18].

De novo lipid synthesis begins with cytosolic citrate, which is converted to acetyl-CoA under the action of ATP-citrate lyase (ACLY), and then catalyzed by acetyl-CoA carboxylase (ACC) to generate malonyl-CoA [19]. Fatty acid synthase (FASN) catalyzes sequential condensation, reduction, dehydration, and again reduction using malonyl-CoA and acetyl-CoA to generate palmitate, which can be further modified by enzymes such as elongation of very long-chain fatty acid proteins (ELOVLs), stearoyl-coA desaturase (SCD), and fatty acid desaturase 2 (FADS2) to produce saturated and monounsaturated fatty acids. Additionally, acetate can also be converted to acetyl-CoA by acetyl-CoA synthetase (ACSS) [20].

Cholesterol is an essential structural component of cell membranes, and its biosynthesis involves the mevalonate pathway. Cholesterol biosynthesis is initiated by the condensation of acetyl-CoA, mediated by acetyl-CoA acetyltransferase (ACAT), producing acetoacetyl-CoA. This intermediate is subsequently converted by 3-hydroxy-3-methylglutaryl-CoA synthase (HMGCS) into HMG-CoA, which is then reduced to mevalonate by HMG-CoA reductase—the rate-limiting step of the pathway. A cascade of downstream reactions eventually generates cholesterol.

Cellular lipids are primarily stored in lipid droplets (LDs) in the form of triacylglycerols (TAGs). LDs are highly mobile and dynamic organelles [21] that regulate lipid release through the inhibition or activation of lipases [22]. Free fatty acids (FFAs) are transported by fatty acid-binding proteins (FABPs). They enter the mitochondria using the carnitine shuttle system. In the mitochondria, FFAs undergo FAO to generate acetyl-CoA, which fuels the tricarboxylic acid (TCA) cycle and oxidative phosphorylation to produce ATP [23].

### 2.2. Enhanced Lipid Uptake and De Novo Synthesis in Pulmonary Hypertension

Fatty acid metabolism encompasses cellular fatty acid uptake and storage, mitochondrial transport, mitochondrial β-oxidation, and de novo fatty acid synthesis. While these processes maintain energy homeostasis and membrane biogenesis under physiological conditions, they are reprogrammed during PH (Figure 1).

#### 2.2.1. De Novo Lipid Synthesis

Enhanced lipid synthesis is a well-established metabolic hallmark of cancer [24]. Recent metabolomic and transcriptomic analyses of PH patients show activation of lipid, nucleotide, and fatty acid metabolism pathways. This pro-proliferative metabolic shift is also observed in PH, where pulmonary vascular cells activate the entire pathway to build new fats de novo (Figure 1) [8,25,26,27,28,29,30,31,32].

The first stage, generating cytosolic acetyl-CoA, is primarily controlled by ATP-citrate lyase (ACLY). Grobs et al. reported that in PASMCs isolated from PAH patients, the expression of ACLY and phosphorylated ACLY (pACLY) is markedly upregulated [26]. This enzyme is critical, as its inhibition reduces acetyl-CoA production and subsequently dampens the expression of downstream lipogenic enzymes like acetyl-CoA carboxylase (ACC), FASN, and SCD1. ACLY is transcriptionally regulated by sterol regulatory element-binding protein (SREBP). Studies have shown that SREBP1 expression is significantly increased in hypoxic human retinal microvascular endothelial cells (HRMECs), potentially driving lipid metabolic reprogramming via activation of the HIF-1α/TGF-β pathway, thereby influencing HRMEC proliferation and migration. Modulating this pathway significantly reduces cell proliferation, migration, and pathological neovascularization across in vitro and in vivo studies [33]. Although research on SREBP1 in PH is limited, targeting SREBP1 or its downstream pathways holds potential as a therapeutic target for vascular proliferative conditions such as PH. Studies indicate that, even in the absence of exogenous lipids, PAH PASMCs maintain lipid accumulation and a hyperproliferative state by activating key lipid synthesis enzymes, underscoring the critical role of endogenous lipid synthesis in driving pathological pulmonary vascular remodeling [25]. Activation of the SIRT7-JNK-Akt axis promotes de novo lipid synthesis, leading to excessive lipid accumulation in PASMCs, and supporting abnormal proliferation, migration, and survival, ultimately exacerbating pathological vascular remodeling.

Increased FASN expression and activity have been detected in lung tissues from monocrotaline (MCT)-induced rats, as well as in hypoxic human pulmonary artery smooth muscle cells (HPASMCs) and endothelial cells (HPAECs), where the enzyme mediates the synthesis of palmitate from malonyl-CoA. Inhibiting FASN reduces palmitate levels and cell proliferation [28,29]. Hou et al. reported that under hypoxic conditions, FASN expression and activity are significantly elevated in the lung tissue of hypoxic pulmonary hypertension (HPH) mice and HPASMCs [30]. Pharmacological inhibition of FASN or gene knockdown activates the PI3K/Akt signaling pathway, thereby reversing hypoxia-induced oxidative stress [30].

SCD is a key enzyme in lipid metabolism, and is responsible for the synthesis of monounsaturated fatty acids (MUFAs). Current research suggests that SCD can reprogram cardiac metabolism and regulate cardiac function [34,35]. Inhibition of SCD1 reduces the proliferation of HL-1 cardiomyocytes [27], attenuates AMP-activated protein kinase (AMPK) activation, and mitigates lipid accumulation during hypoxic and lipotoxic conditions [36]. Cardiac Scd1 overexpression in C57BL/6 mice promotes FASN-driven lipogenesis and SFA accumulation, leading to steatosis and severe heart failure [37], whereas Scd1 loss curtails fatty acid synthesis and uptake, attenuating ceramide formation [38].

Beyond key lipogenic enzymes, substrate supply for de novo lipogenesis is also augmented in PH (Figure 1) [39]. Specifically, multiple glucose transporters (e.g., glucose transporter 1 (GLUT1)) and glycolytic enzymes, including hexokinase II (HK II) and phosphofructokinase, are markedly upregulated [40,41,42,43,44,45]. This enhances the channeling of glycolytic intermediates toward citrate synthesis. Meanwhile, circulating Gln levels are significantly elevated in PH patients [46,47], particularly those with BMPR2 mutations [48]. The coordinated upregulation of glycolytic and glutaminolytic pathways ensures a sustained supply of acetyl-CoA and citrate, meeting the heightened lipogenic demand of hyperproliferative PH cells.

#### 2.2.2. Lipid Uptake

CD36, also known as fatty acid translocase, is a cell surface receptor for fatty acids that facilitates the uptake of extracellular lipids [49,50,51]. Findings in BMPR2 mutant mouse models indicate that increased CD36 protein expression in the RV is a hallmark of lipotoxicity in PAH [52]. Studies show that hypoxia upregulates HIF-1α in PASMCs, thereby increasing CD36 expression. In turn, elevated CD36 activity enhances the cellular capacity for fatty acid uptake, leading to heightened HIF-1α expression and the formation of a “HIF-1α-CD36” positive feedback loop [31]. CD36, as a plasma membrane fatty acid transporter, facilitates the entry of fatty acids into cells. Once inside the mitochondria, fatty acids undergo catalysis by acyl-CoA dehydrogenase to initiate the β-oxidation pathway. Studies have demonstrated that elevated CD36 expression enhances free fatty acid uptake and FAO, thereby providing energy to support cell proliferation and survival [53,54]. However, in PAH, increased CD36-mediated fatty acid uptake, combined with impaired FAO, leads to lipid accumulation, thereby contributing to RV dysfunction and adverse metabolic outcomes [55,56,57]. This paradoxical phenomenon is likely due to excessive fatty acid influx that overwhelms mitochondrial processing capacity under sustained hypoxic or lipotoxic conditions. Consequently, unoxidized lipids accumulate, reactive oxygen species (ROS) generation increases, and autophagic activity is suppressed. Collectively, these maladaptive responses further impair FAO.

### 2.3. Dysregulation of FAO

**Figure 2:**
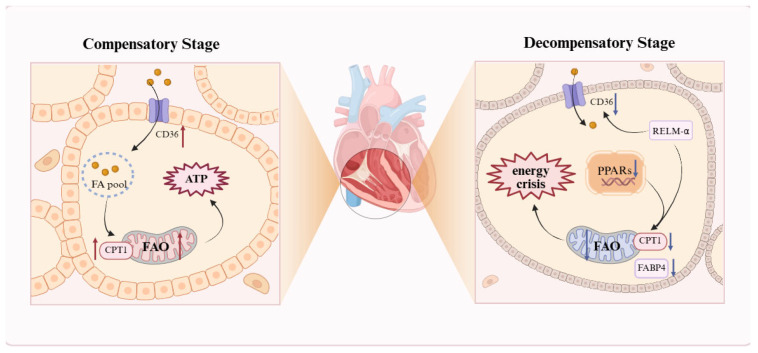
The “double-edged sword” effect of the FAO. Schematic illustration of FAO dysregulation in PH, reflecting the heterogeneity of lipid metabolism. RV cardiomyocytes display stage-dependent changes: during compensation, upregulated CD36 and CPT1 promote FA uptake and oxidation to generate ATP and sustain hypertrophy; during decompensation, reduced PPARγ and CPT1 activity decreases FAO, causing lipid accumulation, lipotoxicity, and an energy deficit. The resulting metabolic shift toward inefficient glycolysis exacerbates RV dilation and failure. A red upward arrow indicates increased activity or expression levels, while a blue downward arrow denotes decreased activity or expression levels. FA: fatty acid; FABP4: fatty acid-binding protein 4; FAO: fatty acid oxidation; PPARs: peroxisome proliferator-activated receptors; RELM-α: resistin-like molecule α. This figure was created with BioRender.com.

**Figure 3:**
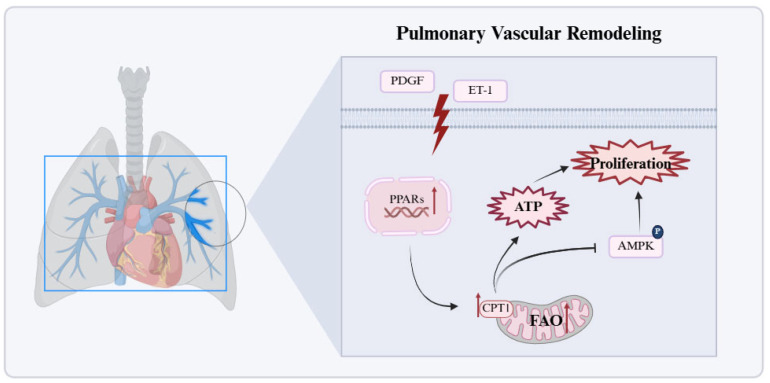
Significant enhancement of FAO in pulmonary vascular cells. In pulmonary vascular cells, persistently increased FAO activates the PPARγ/CPT1 pathway, elevates ATP production, suppresses AMPK signaling, and drives proliferation and remodeling. Red arrows in the figure denote the upregulated expression of target molecules in PH. AMP: activated protein kinase; ET-1: endothelin-1; PDGF: platelet-derived growth factor; PPARs: peroxisome proliferator-activated receptors. This figure was created with BioRender.com.

In PH, FAO serves as a core component of cellular energy metabolism, and is closely associated with right heart failure and pathological remodeling [58]. Fatty acids are shuttled into mitochondria for β-oxidation, yielding acetyl-CoA that feeds into the TCA cycle to support ATP production. FAO facilitates the accumulation of acetyl-CoA in the cytosol, essential for initiating FAS; thus, FAS and FAO can mutually support each other [59]. However, in the pathological environment of PH, this metabolic balance is disrupted, with FAO dysregulation often stemming from progressive mitochondrial dysfunction, thereby amplifying oxidative stress and reprogramming effects [60,61]. This manifests as significant tissue-specific dysregulation: in the RV, FAO undergoes a dynamic evolution from compensatory upregulation to decompensatory downregulation; in pulmonary vessels, FAO exhibits sustained abnormal enhancement, driving the two core pathological processes of right heart failure and vascular remodeling, respectively (Figure 2 and Figure 3).

#### 2.3.1. Bidirectional Dysregulation of FAO in Cardiomyocytes

Cardiac energy metabolism in healthy adults is predominantly supported by FAO, which supplies approximately 60–90% of ATP, with the balance derived from the oxidation of glucose, lactate, amino acids, or ketone bodies [62]. RV dysfunction evolves through stages from compensatory hypertrophy to decompensatory failure, rather than a continuous pathological process. In early PH, the RV adapts to elevated pressure through hypertrophy to maintain function. Increased FAO acts as a compensatory mechanism to meet heightened energy demands and sustain right heart performance. As pressure overload persists, the RV dilates and fails, with reduced FAO leading to lipid accumulation and energy deficiency [63,64,65,66].

In the compensatory phase, the RV maintains cardiac output and functional stability through hypertrophic remodeling (Figure 2). Due to increased ATP demand in cardiomyocytes, studies indicate that expression of the fatty acid transport protein CD36 and the key FAO enzyme carnitine palmitoyltransferase I (CPT1) is elevated, promoting FA uptake and oxidation [67]; thus, increased FAO is considered a core mechanism underlying RV hypertrophy in PAH [68,69,70]. Paulin et al. further revealed that in the MCT rat model, compensatory RV tissue exhibits elevated expression and activity of the transcription factor myocyte enhancer factor 2 (Mef2), closely associated with RV hypertrophy [66]. Mef2 promotes mitochondrial biogenesis by activating PGC-1α [71]; additionally, Yuan et al. found that Mef2 overexpression increases Cpt1b mRNA expression and Cpt1b promoter transcriptional activity [72]. Sustained pressure overload leads to RV dilation and failure. FAO markedly decreases, preventing effective oxidation of fatty acids (FAs). Consequently, FAs accumulate as toxic metabolites, such as ceramides, triggering lipotoxicity and an energy crisis. Recent transcriptomic and multi-omic analyses of PH animal models and human failing RVs consistently show that compared to compensated stages, decompensated RVs exhibit negative enrichment or transcriptional downregulation of nearly all pathways related to fatty acid metabolism and mitochondrial function, indicating a programmatic transcriptional shift beyond isolated enzyme changes [73,74]. For example, Khassafi et al. performed transcriptional profiling in PH models and found that decompensated RVs exhibit distinct molecular subgroups with negative enrichment in fatty acid metabolism and mitochondrial pathways, underscoring a coordinated transcriptional reprogramming toward maladaptive remodeling [75].

The peroxisome proliferator-activated receptor (PPAR) family plays a major role in regulating lipogenesis and glucose metabolism [76,77]. In PAH, reduced mitochondrial FAO in RV cardiomyocytes is primarily due to decreased PPARγ activity and significant downregulation of key FAO genes CPT1b and FABP4, shifting energy metabolism from efficient FAO to inefficient glycolysis, resulting in insufficient ATP production and exacerbating mitochondrial dysfunction [78]. Furthermore, resistin-like molecule α reduces FA entry into mitochondria by inhibiting CPT1a/b and CD36; although glycolysis is increased, energy output is far lower than that of FAO, causing an energy crisis in cardiomyocytes [79]. In hereditary PAH, BMPR2 mutations exacerbate FAO decline, accompanied by abnormal ACC phosphorylation promoting lipid synthesis [50,80]. The WNK1 signaling pathway is further aberrantly activated, inhibiting AMPK and downregulating metabolic enzymes such as CPT1, leading to the accumulation of lipotoxic metabolites and energy metabolic imbalance [52]. In severe PAH rat models, RV fatty acid uptake is reduced by 2.1-fold, with decreased expression of fatty acid transporters and FAO enzymes [81]. Agrawal et al. demonstrated that L-carnitine treatment restores CPT1 activity, improves FAO, and alleviates RV failure [82]. Therefore, current animal models primarily focus on improving RV function by restoring normal FAO and reducing lipid accumulation. In summary, the RV exhibits a unique metabolic program, and distinguishing the stages of RV metabolism in PH is crucial for ensuring the timing and efficacy of treatments.

#### 2.3.2. Significant Enhancement of FAO in Pulmonary Vascular Cells

In contrast to the bidirectional FAO dysregulation in the RV, increased FAO in PH pulmonary vascular cells is a core mechanism driving cell proliferation and metabolic abnormalities (Figure 3) [83]. Pro-proliferative signals such as PDGF and ET-1 upregulate CPT1 expression via activation of the PPARγ/CPT1 pathway, inhibit apoptosis, and promote vascular wall thickening [84]. Additionally, CPT1 protein expression and activity are significantly increased in the lung tissue of MCT-induced PAH rats. CPT1 activation upregulates FAO by reducing malonyl-CoA production, increasing ATP generation, and inhibiting AMPK activation. This further decreases downstream p53 and p21 expression, leading to dysregulated cell cycle control and promoting cell proliferation [85]. Another enzyme involved in FAO, malonyl-CoA decarboxylase (MCD), catalyzes the reaction between malonyl-CoA and acetyl-CoA. MCD inhibition elevates substrate malonyl-CoA levels, thereby reducing FAO, relieving pyruvate dehydrogenase (PDH) inhibition, promoting glucose oxidation (GO), and restoring mitochondrial oxidative phosphorylation [86]. Upregulation of FAO enzyme transcription has been noted in pulmonary arteries from PAH patients, with diverse cell populations such as endothelial, smooth muscle, fibroblastic, and inflammatory cells contributing to disease progression through FAO activation. Importantly, the inhibition of FAO reduces disease severity in experimental models and may benefit PAH patients [87].

Although the modulation of FAO in PH is not completely defined, the reciprocal interplay between FAO and GO, the Randle cycle, remains an important consideration [88]. The activation of one pathway reciprocally suppresses the other. In particular, when glucose oxidation is disrupted, mitochondria maintain energy homeostasis through metabolic reprogramming. In this process, cytosolic pyruvate is converted to FAs, which then enter the mitochondrial matrix via the carnitine shuttle system. Within the mitochondria, FAs undergo β-oxidation to break down into acetyl-CoA, which enters the TCA cycle and ultimately produces ATP through oxidative phosphorylation, compensating for the energy deficit caused by limited glucose utilization. Leveraging this relationship, pharmacological inhibition of FAO with trimetazidine or ranolazine enhances glucose oxidation in PH models and alleviates RV hypertrophy [89]. A randomized, double-blind, controlled trial demonstrated that ranolazine improves RV function in patients with precapillary PH [90]. Trimetazidine also significantly inhibits PASMC proliferation in Su/Hx-induced PH rats and downregulates glycolytic levels in the lungs of experimental rats [91,92].

## 3. Reconstruction of Lipid Signaling Networks

In addition to direct metabolic effects, such as substrate availability and energy reprogramming, the heterogeneity of lipid metabolism in PH is further orchestrated by bioactive lipid signaling networks. These networks act as molecular switches that determine whether lipid accumulation serves as a signal for sustaining cell survival or a trigger for lipotoxicity and cell death. These pathways extend outward to membrane receptors and inward to nuclear epigenetics. In this section, we divide these networks into membrane receptor-mediated signaling for proliferation and inflammation, and intranuclear metabolic signaling for epigenetic regulation. This reconstruction mediates the divergent cellular fates observed in pulmonary vascular remodeling versus right ventricular failure.

### 3.1. Membrane Receptor-Mediated Signaling: Proliferation and Inflammation

**Figure 4:**
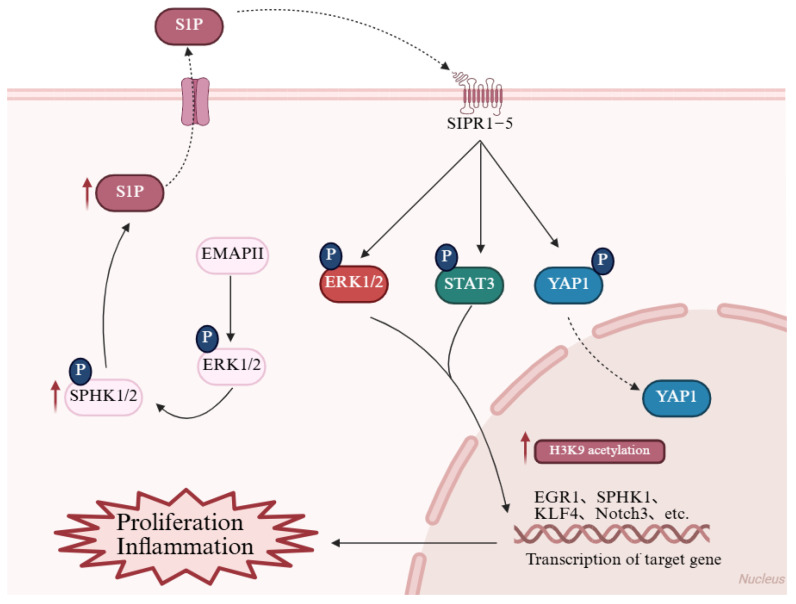
Reconstruction of the sphingolipid signaling network. The bioactive sphingolipid S1P, synthesized by SphK1/2, regulates cell proliferation and inflammation. In PH, elevated S1P levels bind to S1PR1–5 receptors and activate downstream pathways, including the YAP, JAK/STAT, and ERK pathway, thereby promoting the transcription of EGR1, SPHK1, KLF4, and Notch3. These changes ultimately drive cell proliferation and amplify inflammatory responses. Additionally, endothelial-derived EMAP II further triggers the SphK2/S1P axis, specifically inducing histone acetylations in PASMCs, which contribute to pulmonary vascular remodeling. Red arrows in the figure denote the upregulated expression of target molecules in PH. EGR1: Early growth response 1; EMAP II: Endothelial monocyte-activating polypeptide II; ERK1/2: Extracellular signal-regulated kinase 1/2; H3K9: Histone H3 lysine 9; KLF4: Krüppel-like factor 4; Notch3: Notch receptor 3; S1P: Sphingosine-1-phosphate; S1PR1–5 (labeled SIPR1-5 in figure): Sphingosine-1-phosphate receptors 1–5; SPHK1/2: Sphingosine kinase 1/2; STAT3: Signal transducer and activator of transcription 3; YAP1: Yes-associated protein 1. This figure was created with BioRender.com.

Bioactive lipids are potent drivers of the proliferation and inflammation that characterize PH remodeling, largely by activating cell-surface receptor pathways. Sphingolipids are membrane-associated bioactive lipids that serve key functions in cellular signaling and regulatory processes [93,94]. Among them, sphingosine-1-phosphate (S1P) is a critical bioactive lipid synthesized by sphingosine kinases (SphKs) 1 and 2. S1P is critical in driving cellular proliferation, migration, and inflammation. Multiple studies have demonstrated the impact of S1P and its receptors on the development and progression of PAH [95,96,97,98]. Upon tissue injury or hypoxia, intracellular S1P levels rise and are transported “inside-out” to the extracellular space, binding to sphingosine-1-phosphate receptor 1–5 and activating signaling pathways such as YAP [99,100], JAK/STAT [101,102], and ERK [103], inducing cell proliferation, phagocyte recruitment, and secretion of pro-inflammatory cytokines (such as interleukin-1 beta/Interleukin-6, tumor necrosis factor alpha), thereby amplifying inflammatory responses (Figure 4) [104]. Evidence indicates that SphK1 and S1P levels are aberrantly surged in the lungs of PAH patients and HPH animal models. Notably, S1P potently drives PASMC hyperproliferation, triggering a substantial 2.29-fold amplification by 72 h [105]. SphK1 overexpression, possibly via S1PR2 linkage, enhances S1P-induced PASMC proliferation in vitro [97]. SphK1 deficiency or pharmacological inhibition in mice prevents HPH development [99,106], whereas SphK2 deficiency has no such effect. Overall, these studies highlight the “inside-out” nature of S1P signaling, where intracellular production leads to extracellular receptor activation, promoting a pro-proliferative and pro-inflammatory environment in the pulmonary vasculature. Dysregulation of the SphKs/S1P/S1PR axis thus represents a promising therapeutic target for mitigating the membrane-initiated pathological cascades in PH.

### 3.2. Intranuclear Metabolic Signaling: Epigenetic Regulation

The reconstruction of lipid signaling networks in PH extends beyond the cell membrane into the nuclear compartment, establishing a direct crosstalk between metabolic status and epigenetic regulation [107,108]. In this signaling paradigm, lipid metabolic enzymes and their bioactive products directly modulate chromatin architecture and gene expression. This integration of metabolic cues with gene regulation creates a feedback loop that sustains disease progression.

ACLY plays a pivotal role by converting citrate into acetyl-CoA, which serves dual purposes in lipid synthesis and as a donor for histone acetyltransferases. In PAH PASMCs, heightened ACLY activity leads to increased nuclear acetyl-CoA levels, resulting in enhanced histone acetylation that activates genes linked to cell growth and remodeling [26]. Inhibiting ACLY has been found to decrease specific acetylations such as H3K27ac and H4K16ac, thereby downregulating proliferative genes like HIF1A and PDGFB, and reducing vascular changes in experimental models like Sugen/hypoxia rats [26]. Additionally, therapies targeting this pathway show promise in reducing lipid-driven inflammation and vascular remodeling [109].

Nuclear SphK2 contributes by phosphorylating sphingosine to produce S1P, which modulates histone acetylation in the nucleus. In PH, SphK2’s nuclear localization leads to H3K9 hyperacetylation at promoters of genes involved in cell proliferation, such as CCND1 and MYC, exacerbating PASMC remodeling. Studies indicate that SphK2 levels can rise up to 20-fold in idiopathic PAH lung tissues, and its activation by endothelial factors like EMAP II triggers this epigenetic shift, promoting vascular pathology in models like monocrotaline-induced PH [110]. Genetic knockout or pharmacological blockade of SphK2 reduces H3K9 acetylation, mitigating right ventricular overload and disease severity [110]. This pathway also intersects with inflammatory responses. The SPHK2/S1P signaling pathway promotes p53 acetylation by modulating HDAC1/2 activity in acute lung injury, thereby driving NLRP3 transcription and inflammasome activation. Furthermore, studies have revealed that the specific SPHK2 inhibitor Opaganib significantly alleviates lung tissue damage and promotes M2 macrophage polarization. Emerging evidence highlights SphK2’s role in linking ceramide metabolism to epigenetic repression, offering insights into anti-apoptotic mechanisms in vascular cells [111].

In summary, the epigenetic modifications controlled by intranuclear lipid signaling axes, such as ACLY-mediated acetyl-CoA production and SphK2-driven S1P modulation, amplify the heterogeneity of disease effects in PH. These mechanisms generate variable patterns of histone acetylation that influence gene expression in a context-dependent manner. This metabolic-epigenetic interplay generates distinct inflammatory microenvironments across pulmonary compartments and among patient subtypes, thus accounting for the observed heterogeneity in disease severity and therapeutic. Recent advances in single-cell and multi-omics have begun to illuminate these complex landscapes, revealing how distinct epigenetic marks underpin the diverse cellular phenotypes characteristic of pulmonary vascular remodeling [109,112]. Consequently, further integration of these high-resolution omics technologies to decode the spatiotemporal dynamics of the metabolic-epigenetic interface represents a critical priority for future research and the development of precision medicine.

## 4. Cell-Specific Metabolic Phenotypes and Interactions

**Figure 5:**
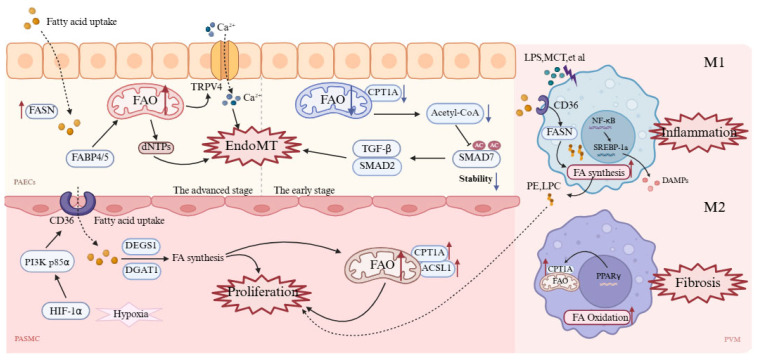
Cell-specific mechanisms of metabolic dysregulation in pulmonary vascular cells during PH. In PAECs, early suppression of FAO via reduced CPT1A lowers acetyl-CoA, activates TGF-β/SMAD2 signaling, and triggers EndoMT. With disease progression, PAECs upregulate FAS for membrane biogenesis, while FABP4/5-driven FAO supports dNTP production and Ca^2+^ overload further promotes EndoMT. In PASMCs, the HIF-1α/PI3K axis upregulates CD36 and CPT1a, enhancing FA uptake and β-oxidation to sustain hyperproliferation and vascular remodeling. In macrophages, M1 cells activate CD36 and FASN to increase lipogenesis and inflammatory mediator release, whereas M2 cells rely on PPARγ/CPT1A-driven FAO for repair, although excessive activation contributes to fibrosis. A red upward arrow indicates increased activity or expression levels, while a blue downward arrow denotes decreased activity or expression levels. ACSL1: Acyl-CoA synthetase long-chain family member 1; CPT1A (labeled CPT1A in M2 cell): Carnitine palmitoyltransferase 1A; DAMPs: Damage-associated molecular patterns; DGAT1: Diacylglycerol acyltransferase 1; dNTPs: Deoxynucleoside triphosphates; EndoMT: Endothelial-to-mesenchymal transition; FABP4/5: Fatty acid-binding protein 4/5; FASN: Fatty acid synthase; HIF-1α: Hypoxia-inducible factor-1 α; LPC: Lysophosphatidylcholine; LPS: Lipopolysaccharide; MCT: Monocrotaline; NF-κB: Nuclear factor-κB; PAECs: Pulmonary artery endothelial cells; PASMC: Pulmonary artery smooth muscle cell; PE: Phosphatidylethanolamine; PI3K p85α: Phosphoinositide 3-kinase p85α; PPARγ: Peroxisome proliferator-activated receptor γ; PVM: Pulmonary Vascular Macrophages; SMAD2/7: Mothers against decapentaplegic homolog 2/7; SREBP-1a: Sterol regulatory element-binding protein-1a; TGF-β: Transforming growth factor-β; TRPV4: Transient receptor potential vanilloid 4. This figure was created with BioRender.com.

The aforementioned dysregulated metabolic phenotypes and mechanisms are not universal across all pulmonary vascular cell types but exhibit cell specificity (Figure 5 and Table 1).

### 4.1. Pulmonary Artery Endothelial Cells

Endothelial-to-mesenchymal transition (EndoMT) is a key pathway through which PAECs participate in PAH pulmonary vascular remodeling [113,114]. During EndoMT, PAECs lose their endothelial phenotype and acquire mesenchymal cell characteristics, including enhanced proliferation, migration, and extracellular matrix deposition capabilities. Early studies have shown that after initial EC apoptosis, surviving antiapoptotic ECs proliferate abnormally to adapt to high energy demands, thereby driving vascular remodeling [115]. Therefore, the upregulation of glycolysis and fatty acid metabolism meets the rapid proliferation demands of ECs. Augmented expression and activity of FASN have been observed in hypoxic human pulmonary artery endothelial cells [28]. Importantly, inhibiting FASN increases endothelial nitric oxide synthase (eNOS) expression in hypoxic HPAECs, promotes apoptosis and glucose oxidation, and reduces cell proliferation, autophagy, and glycolysis levels [28]. Thus, inhibiting FA metabolism in endothelial cells may be a strategy to promote a metabolic shift toward GO. PAH ECs exhibit a profound metabolic shift toward enhanced fatty acid synthesis, thereby regulating the lipid supply necessary for rapid membrane expansion and pathological growth. Moreover, as lipid chaperones, FABP4/5 promote fatty acid influx, upregulate FAO in PAECs and increase dNTP synthesis to support abnormal proliferation [116]. Excessive FAO products (such as β-hydroxybutyrate) can increase TRPV4 channel activity and promote Ca^2+^ influx, leading to mitochondrial dysfunction and endothelial cell injury, with Ca^2+^ overload further triggering EndoMT [117].

However, FAO in pulmonary artery endothelial cells is complex. Some scholars have reported that BMPR2 mutant ECs reduce the number of TCA cycle intermediates, with decreased transcription of enzymes involved in fatty acid metabolism, including the rate-limiting enzyme of FAO—CPT1 [118]. This induces EndoMT through pathways such as AMPK/mTOR and epigenetic regulation [119]. For example, during EndoMT, human pulmonary microvascular endothelial cells (HPMVECs) exhibit reduced CPT1a expression and a significant decline in FAO capacity; mechanistically, FAO inhibition leads to decreased intracellular acetyl-CoA levels, thereby reducing SMAD7 acetylation, lowering its stability and thus enhancing the TGF-β/SMAD2 signaling pathway, promoting EndoMT occurrence [120]. This contradictory phenomenon may occur because normal pulmonary endothelial homeostasis requires FAO. However in early disease stages, downregulating FAO serves as a self-protective mechanism, whereas as the disease progresses, increased energy demands make cells more dependent on FAO, and unable to downregulate it further. Moreover, recent single-cell and spatial transcriptomic analyses of PH models and patients have revealed more important information [121,122]. Upregulated FABP4/5 in pulmonary endothelial cells control fatty acid flux and drive glycolytic reprogramming, with enhanced expression of genes like Eno1, Ldha, and Pkm, promoting vascular remodeling via ROS/HIF-2α signaling [116]. FABP4/5 deletion normalizes endothelial fatty acid metabolism and reverses the pathological accumulation of arterial endothelial cells [116], highlighting that lipid metabolic reprogramming drives vascular remodeling.

### 4.2. Pulmonary Artery Smooth Muscle Cells

The phenotypic transition of PASMCs is a key driver of uncontrolled pulmonary vascular remodeling in PH [123]. Changes in lipid metabolism significantly promote the phenotypic conversion of vascular smooth muscle cells [124], particularly alterations in lipid metabolic enzyme activity [125], which increases FA uptake and β-oxidation, providing energy and substrates for hyperproliferative PASMCs. Studies have shown that in PAH, hypoxia upregulates CD36 expression via activation of the HIF-1α/PI3K p85α axis, promoting PASMC uptake of free FAs, thereby driving abnormal PASMC proliferation and pulmonary vascular remodeling; inhibition of CD36 in animal models significantly alleviates the pathological features of pulmonary hypertension [27]. In the NFU1 gene G206C mutation-induced PAH model, upregulation of the transporters CD36 and CPT1A promotes extracellular FA uptake and mitochondrial transport; Acyl-CoA synthetase long-chain family member 1 upregulation further enhances FAO, maintaining a hyperproliferative phenotype and ultimately driving PAH vascular remodeling [126].

### 4.3. Pulmonary Vascular Macrophages

Macrophages are broadly classified into M1, the classically activated pro-inflammatory type, and M2, the alternatively activated anti-inflammatory type. In PH, an imbalance between M1 and M2 lipid metabolism results in a vicious cycle that drives inflammation and remodeling [127]. M1 macrophages in the PH environment exhibit increased FA synthesis and triglyceride storage. Specifically, under LPS stimulation, M1 macrophages upregulate CD36 expression [57], promoting FA uptake and increasing the activity of the lipid synthesis enzyme FASN, leading to lipid droplet accumulation and the generation of lipid peroxidation products, such as phosphatidylethanolamine and lysophosphatidylcholine [128]. These damage-associated molecular patterns (DAMPs) further activate the complement system, exacerbating M1 pro-inflammatory polarization and driving the synthesis of inflammatory mediators such as prostaglandin E2 via the NF-κB/SREBP-1a pathway [129], ultimately promoting PASMC proliferation and vascular remodeling.

Conversely, M2 macrophages rely primarily on FAO to support oxidative phosphorylation [130], regulating CPT1A expression through PPARγ to promote FAO [131], supporting anti-inflammatory and tissue repair functions. Although M2-type macrophages increase in the late stages of PH, as the M2/M1 ratio increases to nearly 10, promoting vascular remodeling and repair, excessive activation exacerbates fibrosis. Studies have shown that inhibiting M2 macrophage activation in MCT-treated rats effectively reduces PASMC proliferation and improves pulmonary vascular remodeling [132]. Similarly, in semaxanib (SU5416)/hypoxia (SuHx) model rats and PAH patients, perivascular macrophage infiltration increases and exhibits positive staining for FAO enzymes, whereas infiltration decreases after using the CPT1 inhibitor oxfenicine [87,133]. Thus, targeted FAO inhibitors may serve as potential therapeutic targets.

## 5. Targeting Lipid Metabolic Reprogramming in PH Therapeutic Strategies

### 5.1. Targeting Fatty Acid Synthesis

As mentioned earlier, excessive upregulation of fatty acid and cholesterol synthesis directly promotes pulmonary vascular remodeling by providing structural components for biomembranes and energy substrates for the abnormal proliferation of pulmonary vascular cells. Therefore, targeting de novo lipid synthesis is also an attractive therapeutic option (Table 2). Preliminary studies indicate that C75, a specific inhibitor of FASN, can activate PI3K/Akt signaling and regulate mitochondria-dependent apoptosis [26]. In MCT-induced PH models, C75 (2 mg/kg, intraperitoneal injection) significantly reduces right ventricular systolic pressure (RVSP) and right ventricular hypertrophy index (RVHI). This is achieved by reversing the decline in mitochondrial membrane potential, reducing ROS accumulation, enhancing ATP production capacity, and restoring mitochondrial respiratory chain complex function, thereby alleviating pulmonary vascular remodeling and improving right heart function [24,134].

Several natural and synthetic inhibitors of ACLY have been identified, many of which are in use in cancer clinical studies. Among them, the pharmacological inhibitor of ACLY, BMS-303141, reduces PASMC proliferation, migration, and apoptosis resistance by inhibiting de novo fatty acid synthesis and histone acetylation, reversing metabolic reprogramming and epigenetic abnormalities [26]. In Sugen/hypoxia rat models, BMS-303141 substantially lowers RVSP (*p* < 0.001), alleviates pulmonary vascular remodeling, and demonstrates a favorable safety profile [26]. Therefore, targeting ACLY offers a multidimensional therapeutic strategy for PAH that combines metabolic and epigenetic regulation.

Another feasible approach is to target key upstream regulatory factors to achieve more comprehensive control of lipid synthesis. In hereditary pulmonary arterial hypertension, BMPR2 mutations induce RV lipotoxicity, manifesting as FAO defects, triglyceride and ceramide deposition, leading to impaired RV hypertrophy and heart failure [50]. Metformin reprograms lipid metabolism by activating the AMPK pathway, inhibiting lipid synthesis enzymes, and reducing RV triglyceride deposition [68]. Furthermore, in an 8-week single-center phase II trial (NCT01884051) conducted in patients with idiopathic or hereditary PAH, metformin was safe, well tolerated, and effectively reversed PH-induced lipotoxicity and vascular remodeling [135].

### 5.2. Targeting Fatty Acid Oxidation

In addition to abnormalities in lipid synthesis, FAO dysregulation plays a crucial role in providing energy for pulmonary vascular cell proliferation, making the inhibition of FA utilization in pulmonary vascular cells a potential therapeutic strategy for PH (Table 2). Among these, CPT1, the key rate-limiting enzyme in mitochondrial FAO, represents a pharmacological target for FAO inhibition. Oxfenicine prevents and partially reverses SuHx-induced PH in mice, alleviating pulmonary vascular constriction and remodeling [87]. Another CPT1 inhibitor, etomoxir (ETO), promotes p53 and p21 expression by activating AMPK. This induces cell cycle arrest at the G2/M phase and inhibits PASMC proliferation in MCT model rats [85]. Perhexiline, a recognized CPT1 inhibitor commonly used for heart failure treatment [136], and its fluorinated derivative fluorinated perhexiline derivative 1 (FPER-1), reverse the Warburg effect in PASMCs of PAH by inhibiting CPT1 [84]. However, research on CPT1 inhibitors for PH treatment remains in the early stages, and extensive clinical trials are needed to evaluate the efficacy and safety of such drugs.

Trimetazidine (TMZ) and ranolazine, as partial FAO inhibitors, restore PDH activity and GO levels by reducing FAO in rat models of RV hypertrophy, thereby increasing ATP levels, cardiac output, and the treadmill distance in rats [89]. TMZ, an inhibitor of the mitochondrial enzyme long-chain 3-ketoacyl-CoA thiolase, activates the Randle cycle to enhance GO and inhibit FAO, commonly prescribed for the treatment of angina [137], myocardial infarction [138], and heart failure [139,140]. Studies have shown that TMZ improves PH by downregulating the gene expression of fatty acid transporters (such as Cd36, Cpt1a/Cpt2) and glycolytic enzymes in the lung tissue, activating the Randle cycle to promote glucose oxidation, and optimizing energy metabolism [91]. In a phase II clinical trial (NCT02102672) evaluating TMZ as a FAO inhibitor on RV function and remodeling in PAH patients, the results showed that after 3 months of TMZ treatment, the 6 min walk distance significantly increased, with modest improvement in the RV diastolic area, and it was well tolerated, with no significant difference in adverse events compared to the placebo [141].

Unlike TMZ, ranolazine does not directly affect FAO-related enzymes but mitigates cardiomyocyte remodeling through indirect regulation of the pulmonary vasculature, for example, by inhibiting the late sodium current, reducing calcium overload, and preventing electrical remodeling [142]. Ranolazine also increases O_2_ consumption and ATP production, downregulates glycolytic enzymes (such as Glut1, hexokinase I (HKI), and lactate dehydrogenase A (LDHA)), and reduces lactate production [89]. Preclinical studies have demonstrated that ranolazine improves right atrial remodeling in MCT-induced PH rat models [143]. In multiple completed clinical trials of ranolazine in PAH patients (NCT01174173, NCT01757808), it was shown to be safe and well tolerated [144,145], and improved symptoms and echocardiographic parameters in PAH patients [125]. Boston University conducted a phase IV clinical trial to assess the safety and efficacy of ranolazine in patients with PH secondary to left ventricular diastolic dysfunction (NCT02133352), with follow-up after a 6-month treatment period showing significant mPAP decline in subjects. The University of Pennsylvania sponsored a randomized, double-blind, placebo-controlled, multicenter phase IV trial assessing the impact of ranolazine on PH and RV dysfunction (NCT01839110 and NCT02829034) [146]. Another randomized, double-blind, placebo-controlled trial reported that ranolazine improved RV function in patients with precapillary pulmonary hypertension [90], although its therapeutic efficacy still requires confirmation in larger studies.

However, data also suggest that enhancing FAO is a potential therapeutic approach. The PPARγ agonist pioglitazone has significant therapeutic effects on PAH [147,148,149]. Studies have shown that pioglitazone upregulates FAO-related genes through transcriptional regulation, restores RV mitochondrial morphology and function. In the SuHx rat model, oral pioglitazone treatment completely alleviates severe PAH and vascular remodeling and safeguards against RV failure [78]. These findings emphasize the central role of PPARγ in lipid homeostasis and energy metabolism. Although an early phase II trial was terminated due to concerns regarding insulin resistance, its safety and efficacy in chronic lung disease-related PH are under investigation in an ongoing clinical trial (NCT06336798), highlighting the prospects for preclinical-to-clinical translation [150].

In combination, inhibiting or enhancing FAO in PH therapeutic strategies may appear contradictory but actually represents complementary approaches targeting different pathological dimensions of PH. Integrating the analysis of the aforementioned studies, strategies targeting FAO present a significant therapeutic paradox. On the one hand, CPT1 inhibitors (such as Etomoxir) and partial FAO inhibitors (such as Trimetazidine) demonstrate potential in alleviating PH; on the other hand, FAO activators (such as Pioglitazone) also exhibit clear efficacy in reversing PH and RV failure. This phenomenon profoundly reflects the tissue-specific heterogeneity of lipid metabolic reprogramming in the pathophysiology of PH. The theoretical foundation of the FAO-inhibiting strategy lies in the pathological changes of pulmonary vasculature: in PH pulmonary vascular cells (especially PASMCs), FAO undergoes sustained pathological enhancement, providing key energy and biosynthetic precursors for cellular hyperproliferation. Therefore, the therapeutic logic of inhibiting this pathway is to restrict the metabolic fuel supply for vascular remodeling.

In contrast, the FAO-enhancing strategy focuses on the decompensated right ventricle. Unlike the pulmonary vasculature, the failing RV undergoes a pathological downregulation of FAO in cardiomyocytes during the transition from the compensatory phase to the decompensatory phase. This metabolic suppression leads to mitochondrial dysfunction, energy crisis, and accumulation of lipotoxic metabolites (such as ceramides). Therefore, the therapeutic logic of using activators like Pioglitazone is to restore impaired FAO in the myocardium, aiming to rebuild RV energy homeostasis, clear lipotoxic deposits, and alleviate RV hypertrophy and heart failure.

The opposing metabolic profiles between the pulmonary vasculature and right ventricle constitute a core challenge in the clinical translation of current PH metabolic therapies [151]. Non-selective systemic FAO inhibitors, while suppressing pulmonary vascular remodeling, may theoretically exacerbate RV energy depletion and lipotoxic injury. Conversely, systemic FAO activators, although potentially rescuing the failing RV, carry the risk of promoting pathological proliferation in pulmonary vascular cells. Therefore, future precision interventions must pursue tissue-specific regulation. Developing novel drug delivery systems (such as lung-targeted nanoparticles) to achieve differentiated metabolic interventions for the pulmonary vasculature and right ventricle is a key breakthrough in balancing metabolic demands across different tissues and maximizing therapeutic benefits [152].

### 5.3. Therapeutic Potential of Targeting Fatty Acid Transport Proteins

CD36, a cell-surface protein, mediates the uptake of extracellular FAs and transduces signals to multiple downstream effectors. Elevated CD36 expression enhances the cellular uptake of free FAs and promotes FAO, providing energy support for cell proliferation [53]. In tumors and chronic obstructive pulmonary disease, CD36 inhibitors have shown potential in improving disease progression by suppressing lipid uptake and metabolic reprogramming [153,154]. Although clinical trials directly targeting PH are currently limited, CD36-targeted therapies represent a promising direction that warrants further preclinical and clinical investigation.

### 5.4. Prospects of Natural Compounds in PH Treatment

Osthole, a natural coumarin compound derived from the Chinese herb Cnidium monnieri, has significant anti-inflammatory effects on pulmonary diseases [155,156], particularly by alleviating airway hyperresponsiveness in asthma models [157]. Recent studies suggest that osthole may regulate PASMC proliferation [158], apoptosis [159], and metabolic imbalances in PAH rats [160,161]. Niu et al. reported that osthole modulates PASMC proliferation and vascular remodeling by targeting miRNA-22-3p to regulate lipid metabolic reprogramming [160]. Yao reported that osthole effectively reverses elevated S1P levels by regulating the metabolic enzyme SphK1 in PAH rats [161], providing new insights for metabolism-targeted interventions in PH.

Rhodiola, a unique herb from the Tibetan region of China, is used to treat hypoxic symptoms at high altitudes. Modern pharmacological studies have revealed that Rhodiola extracts possess broad anti-inflammatory, antioxidant, antihypertensive, and cardiovascular protective bioactivities [162,163,164]. Its extract, salidroside (Sal), has been found to alleviate HPH by restoring TWIK-related acid-sensitive K+ channel 1 (TASK-1) function [165]. Accumulating evidence indicates Rhodiola’s benefits for metabolic disorders [166,167]. Ren et al. reported that Rhodiola water extract (RCE) significantly improves hemodynamics and cardiac function in PAH rats by inhibiting CPT1a and reducing autophagy through the suppression of PPARγ and LC3B [168].

## 6. Challenges and Future Directions

Although the therapeutic prospects are promising, targeting lipid metabolic reprogramming in PH remains fraught with challenges, primarily stemming from its heterogeneity across tissues, cells, and temporal stages. For instance, excessive FAO in pulmonary vascular cells supplies acetyl-CoA and NADH, thereby driving aberrant proliferation and exacerbating oxidative stress and lipid peroxidation, whereas FAO inhibition can mitigate remodeling. In the RV, FAO undergoes a dynamic shift from compensatory upregulation to decompensatory downregulation, such that indiscriminate inhibition or enhancement may aggravate lipotoxicity and energy deficit [169]. This underscores the risks of non-selective therapies, which may benefit one compartment while worsening another. Zhao et al. utilized dynamic [18F]FDG-PET imaging to quantify glucose uptake and metabolism in a cohort of PH patients, confirming increased pulmonary glucose metabolism in IPAH [170]. Lundgrin et al. demonstrated that fasting FDG-PET provides mechanistic insights into RV failure in PAH patients, with elevated glucose utilization serving as a marker of metabolic stress in the failing RV [171]. In heart failure research, PET with [18F]FTHA and FDG enables quantitative assessment of myocardial free fatty acid and glucose utilization, where [18F]FTHA directly images fatty acid uptake and β-oxidation [172]. Notably, FDG-PET has been integrated into clinical practice for other diseases [173]. In the future, this imaging modality may evolve into a novel tool for monitoring spatiotemporal disruptions in lipid metabolism during compensatory RV hypertrophy versus decompensatory RV failure. Marsboom et al. reported a close correlation between 18FDG uptake in lung tissue and pulmonary vascular remodeling in the SUHx rat model [174]. However, adverse pulmonary vascular remodeling in PH involves multiple cellular components, however, FDG-PET imaging lacks the ability to discriminate these cell types. Therefore, future efforts must leverage single-cell metabolomics to elucidate the spatiotemporal dynamics of lipid metabolism and develop tissue- and cell-specific therapeutic strategies.

In prospective precision interventions, spatially targeted drug delivery technologies represent a highly promising direction [175]. Nanoparticles (NPs) have been employed as innovative drug delivery systems to transport therapeutics to target organs. Li et al. [176] utilized a pulmonary artery-targeted co-delivery system to administer paclitaxel and caspase-3, alleviating MCT-induced PH. Deng et al. [177] developed poly (β-amino) ester nanoparticles for efficient and specific nucleic acid delivery to pulmonary microvascular endothelial cells, with in vivo imaging system whole-body imaging and flow cytometry confirming functional DNA plasmid activity exclusively in lung endothelial cells. These studies suggest that combining drug delivery with metabolic intervention strategies may constitute a key breakthrough for advancing precision PH therapy.

## 7. Conclusions

In summary, alterations in lipid metabolism have become an indisputable component of the metabolic landscape in PH. Here, we discussed emerging lipid metabolic mechanisms that support PH progression, potential lipid-related therapeutic targets, and investigational strategies to attenuate vascular remodeling. Recent studies have established a pathological loop linking metabolism and epigenetics, highlighting the central role of the “lipid signaling–histone acetylation” axis in PAH. ACLY, a key node connecting de novo lipogenesis with histone acetylation, is upregulated in remodeled vessels from both clinical samples and animal models. Pharmacological inhibition of ACLY not only suppresses the expression of lipogenic enzymes and reduces lipid droplet accumulation, but also reshapes the histone acetylation landscape. This limits smooth muscle cell proliferation and vascular remodeling, demonstrating clear therapeutic potential. Similarly, the SphK2/S1P axis contributes to remodeling by modulating histone acetylation in PASMCs. Nuclear SphK2 regulates H3K9 acetylation to promote proliferation, while genetic deletion or pharmacological blockade of this axis significantly attenuates vascular remodeling and RV overload in experimental PH, suggesting that the lipid signaling–epigenetic interface may represent a new entry point for metabolic intervention.

Currently, the preclinical efficacy of therapies targeting lipid metabolism has been extensively validated in Group 1 PAH models. Building on these mechanistic insights, several lipid metabolism targets show strong clinical potential, with their relevance often depending on the stage of PAH progression. In early stage PAH, where pulmonary vascular remodeling and metabolic shifts toward fatty acid synthesis and FAO are prominent, inhibitors of de novo lipogenesis such as ACLY antagonists (e.g., BMS-303141) hold particular promise. These agents suppress PASMC proliferation and epigenetic dysregulation, demonstrating efficacy in preclinical models and early human trials. In mid-to-late stage disease, characterized by right ventricular decompensation and lipotoxicity, partial FAO inhibitors like trimetazidine help improve RV energetics and function by shifting substrate utilization toward glucose oxidation—a benefit supported by recent randomized controlled evidence showing enhanced RV ejection fraction and reduced hypertrophy in PAH patients. Conversely, in advanced RV failure, PPARγ agonists such as pioglitazone can restore mitochondrial FAO and alleviate energy deficits. Of particular note, the clinical trial investigating pioglitazone for Group 3 PH (chronic lung disease-associated pulmonary hypertension) has now entered the implementation phase. This advancement paves a new path for personalized treatment across different pulmonary hypertension subtypes.

Despite this promise, the therapeutic targeting of lipid metabolic reprogramming remains constrained by tissue-, cell-, and stage-specific heterogeneity. Precision strategies that balance efficacy and safety across different disease compartments are essential. Advances in single-cell metabolomics and spatially targeted drug delivery now offer exciting avenues for cell- and tissue-specific interventions. With continued refinement of small-molecule inhibitors and targeted delivery systems, lipid-based therapeutic strategies hold considerable potential for clinical translation. Future efforts should focus on biomarker-guided patient stratification and validation in larger trials to ensure efficacy across diverse PH subtypes [178,179,180].

## Figures and Tables

**Table 1:** Metabolic heterogeneity across major cell types in pulmonary hypertension.

Cell Type	Key Metabolic Features	Functional Consequences	Key Enzymes/Proteins	Pathways
Right Ventricular Cardiomyocytes	Bidirectional FAO Dysregulation: upregulation in compensatory stage; Decompensated stage: FAO downregulation, lipid deposition.	Compensatory stage: Maintains energy supply. Decompensated stage: Energy crisis, lipotoxicity, and cardiac failure.	compensatory stage: ↑CD36, CPT1b, Mef2,PGC-1α Decompensated stage: ↓PPARγ, CPT1b, FABP4	AMPK, WNK1
Pulmonary artery endothelial cells	Paradoxical FAO: Downregulated in early disease or specific mutations; may be upregulated in late stages to meet energy demands. Enhanced fatty acid synthesis.	EndoMT Aberrant proliferation Ca^2+^ overload and mitochondrial dysfunction	↑FASN, FABP4/5 ↓CPT1a	AMPK/mTOR, TGF-β/SMAD2
Pulmonary artery smooth muscle cells	Markedly enhanced fatty acid uptake and FAO, providing energy and biomass for proliferation.	Aberrant proliferation, migration, and apoptosis resistance, driving vascular remodeling.	↑CD36, CPT1A, ACSL1	HIF-1α/PI3K, PPARγ/CPT1
Pulmonary Vascular Macrophages	M1 (Pro-inflammatory): Increased fatty acid synthesis and lipid droplet storage. M2 (Anti-inflammatory): Enhanced FAO supporting oxidative phosphorylation.	M1: Drives inflammation, promotes PASMC proliferation. M2: Anti-inflammatory, tissue repair, but excessive activation exacerbates fibrosis	M1: ↑CD36, FASN M2: ↑CPT1A	NF-κB/SREBP-1a

The upward arrow indicates increased expression; The downward arrow indicates decreased expression.

**Table 2:** Emerging drugs that target lipid metabolism in PH.

Drug	Pathways	Target	Function	Research Subjects	Clinical Trials
Trimetazidine	FAO	Long-chain 3-ketoacyl-CoA thiolase	Reduces FAO, promotes glucose oxidation and improves RV function and remodeling	PAH	NCT02102672
Ranolazine	FAO	Late sodium channel and FAO pathway	Inhibition of FAO, increased glucose utilization, reduced ROS, reversal of RV lipotoxicity and inflammation	PAH	NCT01174173, NCT01757808, NCT01839110, NCT02829034, NCT02133352
Pioglitazone	FAO	PPARγ	Activation of PPARγ, promotion of balanced FAO, reduction of inflammation and remodeling, restoration of mitochondrial morphology and function	PH due to Chronic Lung Disease (CLD)	Currently recruiting clinical trial, NCT06336798
Oxfenicine	FAO	CPT-1	Block fatty acid entry into mitochondria, shift to glucose pathway, reduce lipotoxicity	SuHx rats; Schistosoma and hypoxia-induced PH mice	Preclinical
Etomoxir	FAO	CPT-1	Inhibit FAO, promote glucose utilization, reduce lipid accumulation	MCT rats	Preclinical
Perhexiline	FAO	CPT-1	Inhibit CPT-1, reduce lipid oxidation, promote glucose oxidation, reverse PH cell proliferation	Human PASMCs from PAH doners	Preclinical
FPER-1	FAO	CPT-1,PDH	Fluorinated perhexiline derivative, inhibits FAO, reduces vascular smooth muscle cell proliferation	Human PASMCs from PAH doners	Preclinical
Metformin	FAS	AMPK and fatty acid synthesis pathway	Activate AMPK, inhibit lipid synthesis, reduce lipid accumulation, improve endothelial function and vascular remodeling	PAH	NCT01884051
C75	FAS	FAS	Inhibit fatty acid synthase while increasing FAO, reduce lipid accumulation	Hypoxia-induced PH mice; MCT rats	Preclinical
BMS-303141	FAS	ACLY	Inhibit ACLY, reduce lipid synthesis, reverse vascular remodeling	SuHx rats and mice	Preclinical
Osthole	NA	miRNA-22-3p and lipid metabolism–related enzymes	Regulate miRNA-22-3p, inhibit CD36, FAS, and CPT1A activities, restore lipid homeostasis, and reduce PASMC proliferation and vascular remodeling	MCT rats	Preclinical
Rhodiola crenulata extract	NA	lipid metabolism–related enzymes	Downregulate CPT1A mRNA and protein expression to inhibit FAO; reduce autophagy via PPARγ and LKB1-AMPK signaling pathways	MCT rats	Preclinical

## Data Availability

No new data were created.

## Abbreviations

The following abbreviations are used in this manuscript:

ACAT	Acetyl-CoA acetyltransferase
ACLY	ATP-citrate lyase
ACC/ACACA	Acetyl-CoA carboxylase
ACSS	Acetyl-CoA synthetase
AMPK	AMP-activated protein kinase
BMPR2	Bone morphogenetic protein receptor type 2
CD36	Cluster of differentiation 36
CPT1	Carnitine palmitoyltransferase 1
dNTP	Deoxynucleoside triphosphate
DGAT	Diacylglycerol acyltransferase
EC	Endothelial cell
EMAP II	Endothelial monocyte-activating polypeptide II
EndoMT	Endothelial-to-mesenchymal transition
ERK	Extracellular signal-regulated kinase
ETO	Etomoxir
ET-1	Endothelin-1
FA	Fatty acid
FAO	Fatty acid oxidation
FAS	Fatty acid synthesis
FASN	Fatty acid synthase
FABP	Fatty acid-binding protein
FADS2	Fatty acid desaturase 2
FPER-1	Fluorinated perhexiline derivative 1
GO	Glucose oxidation
HIF-1α	Hypoxia-inducible factor-1 alpha
HMGCS	3-hydroxy-3-methylglutaryl-CoA synthase
HPH	Hypoxic pulmonary hypertension
HPAEC	Human pulmonary artery endothelial cell
HPASMC	Human pulmonary artery smooth muscle cell
HPMVEC	Human pulmonary microvascular endothelial cell
HRMEC	Human retinal microvascular endothelial cell
IPAH	Idiopathic pulmonary arterial hypertension
LD	Lipid droplet
LDHA	Lactate dehydrogenase A
LPS	Lipopolysaccharide
MCT	Monocrotaline
MCD	Malonyl-CoA decarboxylase
Mef2	Myocyte enhancer factor 2
MUFA	Monounsaturated fatty acid
PAEC	Pulmonary artery endothelial cell
PAH	Pulmonary arterial hypertension
PASMC	Pulmonary artery smooth muscle cell
PDH	Pyruvate dehydrogenase
PH	Pulmonary hypertension
RV	Right ventricle
RVSP	Right ventricular systolic pressure
RVHI	Right ventricular hypertrophy index
Sal	Salidroside
S1P	Sphingosine-1-phosphate
SCD	Stearoyl-CoA desaturase
SMAD	Mothers against decapentaplegic homolog
SFA	Saturated fatty acid
SphK1/2	Sphingosine kinase 1/2
SREBP	Sterol regulatory element-binding protein
SuHx	Sugen/hypoxia model
TASK-1	TWIK-related acid-sensitive K+ channel 1
TAG	Triacylglycerol
TCA cycle	Tricarboxylic acid cycle
TGF-β	Transforming growth factor-beta
TMZ	Trimetazidine
